# Non-Stationary Platform Inverse Synthetic Aperture Radar Maneuvering Target Imaging Based on Phase Retrieval

**DOI:** 10.3390/s18103333

**Published:** 2018-10-05

**Authors:** Hongyin Shi, Saixue Xia, Qi Qin, Ting Yang, Zhijun Qiao

**Affiliations:** 1 School of Information Science and Engineering, Yanshan University, Qinhuangdao 066000, China; shihy@ysu.edu.cn (H.S.); 18230330575@163.com (S.X.); 18712786006@163.com (Q.Q.); yang__ting@yeah.net (T.Y.); 2 School of Mathematical & Statistical Sciences, The University of Texas Rio Grande Valley, Edinburg, TX 78539, USA

**Keywords:** inverse synthetic aperture radar (ISAR), non-stationary platform, maneuvering target, autofocus, phase retrieval, oversampling smoothness (OSS)

## Abstract

As a powerful signal processing tool for imaging moving targets, placing radar on a non-stationary platform (such as an aerostat) is a future direction of Inverse Synthetic Aperture Radar (ISAR) systems. However, more phase errors are introduced into the received signal due to the instability of the radar platform, making it difficult for popular algorithms to accurately perform motion compensation, which leads to severe effects in the resultant ISAR images. Moreover, maneuvering targets may have complex motion whose motion parameters are unknown to radar systems. To overcome the issue of non-stationary platform ISAR autofocus imaging, a high-resolution imaging method based on the phase retrieval principle is proposed in this paper. Firstly, based on the spatial geometric and echo models of the ISAR maneuvering target, we can deduce that the radial motion of the radar platform or the vibration does not affect the modulus of the ISAR echo signal, which provides a theoretical basis for the phase recovery theory for the ISAR imaging. Then, we propose an oversampling smoothness (OSS) phase retrieval algorithm with prior information, namely, the phase of the blurred image obtained by the classical imaging algorithm replaces the initial random phase in the original OSS algorithm. In addition, the size of the support domain of the OSS algorithm is set with respect to the blurred target image. Experimental simulation shows that compared with classical imaging methods, the proposed method can obtain the resultant motion-compensated ISAR image without estimating the radar platform and maneuvering target motion parameters, wherein the fictitious target is perfectly focused.

## 1. Introduction

ISAR imaging has been the focus of many researchers and operational users in the last few decades. ISAR imagery plays an important role especially in military applications such as target identification, recognition, and classification [1,2,3,4,5,6]. For ground-based radars, a perspective blind zone will exist when the height of the measured target is too low. In this case, the aerostat radar platform effectively handles the above difficulties and plays an important role in the military field. In comparison with the traditional ISAR system, the aerostat borne radar can utilize the rich spatial resources of the stratosphere. For this reason, combined with the advantages of the aerostat borne radar platform [7] (such as the high anti-stealth effect in the high-altitude survey), it is the focus of research now and in the future. Currently, the basis of the aerostat ISAR imaging is that the radar platform does not move vertically or horizontally. Once the aerostat borne radar platform is affected by stratospheric airflow, etc., however, the displacement or vibration of the platform is unavoidable, that is, the platform is quasi-stationary, which will bring more difficulties in the processing of the received signals.

When the radar platform is in a quasi-stationary state, its motion is random, which greatly increases the difficulty of estimating the motion parameters of the radar platform. In addition, the relative motion between the radar platform and the target is not only caused by the motion of the radar platform, but also the target motion, which makes the motion compensation procedure more complicated. In order to overcome the ISAR image distortion under such conditions, GPS+INS navigation systems have widely been applied [8]. Unfortunately, when the platform carries more devices, it will increase the load on the ISAR system, which is not conducive to the extension of the platform’s service life. The traditional Range-Doppler algorithm [9,10] is usually adopted to produce a 2D ISAR image for slow moving targets in the case of a stationary radar platform. Moreover, as a common method, estimating the motion parameters of the maneuvering target based on the ISAR echo data has some advantages. For example, the maximum probability estimate [11] shows good performance when performing motion parameter estimation, but the amount of calculation of the method is large because it is necessary to search for the estimated values at multiple dimensions. An ISAR imaging motion compensation technology based on parameter estimation is studied in [12,13,14]. References [15,16,17] proposed a cross-correlation method with a small scope of measuring velocity and a minimum entropy method with a large computational cost. Li, et al. [18] combined the Keystone Transform with Fractional Fourier Transform to reduce the computational complexity and increase the accuracy of the compensation. The image contrast-based autofocus (ICBA) mentioned in reference [19] aims to form well-focused ISAR images by maximizing the image contrast (IC), which is an indicator of the image quality. Although the method has a good performance in estimation of the focusing parameters, the process of performing the estimation is too complicated and easy to introduce errors. A compensation method based on the envelope correlation method is applied in the stratosphere ISAR system to compensate for the phase error along the down-range due to the platform drift [20]. However, the above method is also based on parameter estimation of the echo signal.

In recent years, phase retrieval has become a very popular research field [21,22,23,24,25,26]. Since phase retrieval can be applied in many fields such as electron microscopy, crystallography, astronomy, optical imaging, and holographic imaging, its study has attracted considerable attention. The principle of phase retrieval is to recover the original signal only by using amplitude measured of the signal in a certain transform domain (usually the amplitude spectrum of the Fourier transform). Phase retrieval algorithms can roughly be divided into two categories [21]: alternating projection and the other one based on sparse representation. The hybrid-input output (HIO) algorithm [26] and the oversampling smoothness (OSS) algorithm [27] belong to classical alternative projection algorithms. Schniter and Rangan [28] proposed a compressed phase retrieval algorithm to recover sparse signals, that is, using a small amount of data to reconstruct the object. In aerostat-borne ISAR imaging, the random motion of the radar platform or the electromagnetic wave propagation effect in a random medium may affect the phase synchronization of the echo data. By this way, the ISAR autofocus process can be considered as the phase retrieval problem, that is, the error phase information is restored to the correct phase information. In addition, from the spatial geometry model and the imaging mechanism of ISAR, the ISAR echo can be regarded as the Fourier transform of the spatial target. In optical imaging, only the measured Fourier amplitude can be used to performing the phase retrieval. In contrast, in the processing of the ISAR received signal, both the amplitude and error phase (priori information) of the maneuvering target can be utilized.

From the above analysis, based on the imaging mechanism of the aerostat borne ISAR, combined with the phase retrieval principle and priori phase information of the classical imaging algorithm, we propose an ISAR autofocusing imaging method based on the improved OSS algorithm in this paper. The unwanted effects due to the quasi-static state of the aerostat borne radar platform and the target’s motion can be eliminated after applying the proposed method.

The organization of this paper is as follows: Section 2 mainly establishes and analyzes the ISAR non-stationary platform model. In Section 3, we introduce the non-stationary platform ISAR imaging based on the improved phase retrieval algorithm. Experimental results and simulation analysis are presented in Section 4. Conclusions are given in Section 5.

## 2. Non-stationary Platform ISAR Imaging Analysis

### 2.1. ISAR Imaging Geometry Model and Echo Analysis of Platform Displacement

**Figure 1:**
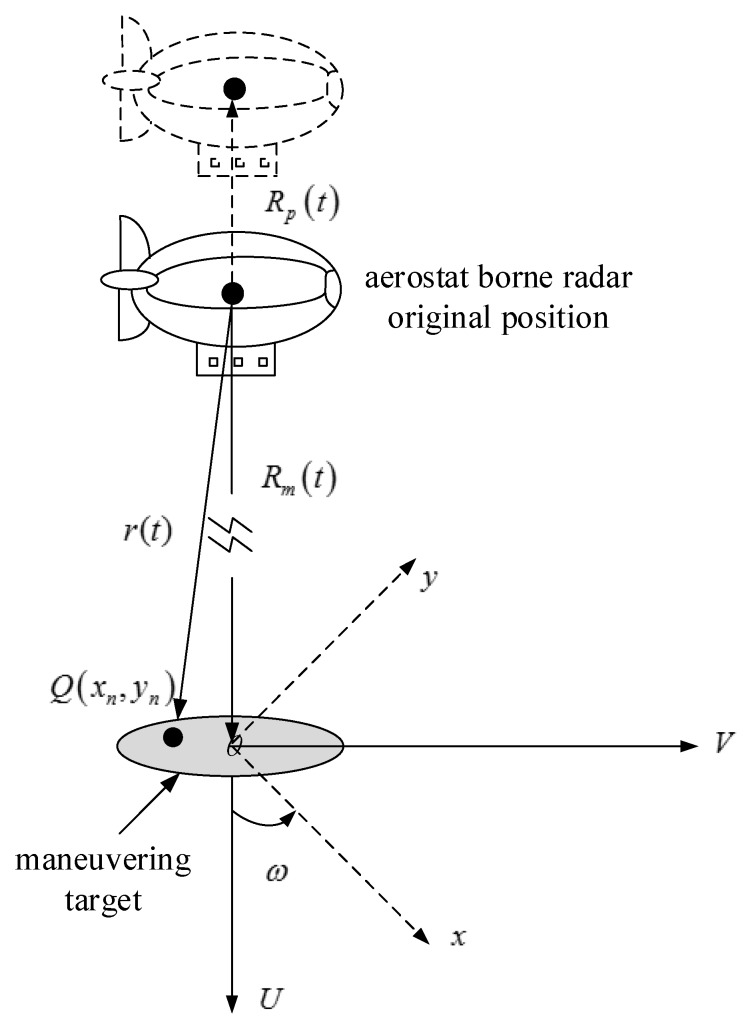
Aerostat borne ISAR imaging geometry model.

Figure 1 shows the aerostat borne ISAR spatial geometry model where *R_p_*(*t*) is the displacement of the radar platform. It is assumed that shaded area represents the maneuvering target and *Q*(*x_n_*,*y_n_*) is a point scatterer on the maneuvering target. The target has radial motion and rotational motion. The maneuvering target rotates around the origin of the *U − V* plane at a uniform angular velocity ω.

For far-field radars, the distance between the scattering point *Q* on the target and the radar platform can be approximated as [11]:

(1) $$r(t)≅R_{m}(t)+R_{p}(t)+x_{n}\cos\omega t-y_{n}\sin\omega t$$

where *R_m_*(*t*) represents the distance from the initial position of the radar to the geometric center *O* of the target and *R_p_*(*t*) is the radial motion of the radar platform. *R_m_*(*t*) is expanded with a Taylor series as:

(2) $$R_{m}(t)=R_{o}+v_{t}t+\frac{1}{2}a_{t}t^{2}+\cdots$$

Here $v_{t}$ and $a_{t}$ are the target’s radial velocity and acceleration, respectively, and *R*_0_ denotes the original distance from the radar to the origin *O*.

For simplification, (2) can be expressed by the following equation:

(3) $$R_{m}(t)=R_{o}+v_{t}t+\frac{1}{2}a_{t}t^{2}$$

In addition, *R_p_*(*t*) can be written as:

(4) $$R_{p}(t)=v_{p}t+\frac{1}{2}a_{p}t^{2}$$

where $v_{p}$ and $a_{p}$ are the radial velocity and acceleration of the radar platform, respectively.

If the ωt is small in a relatively short period of time, so we have:

(5) $$\begin{array}{l} \cos\omega t≅1\\ \sin\omega t≅1 \end{array}$$

In this way, the distance r(t) from the scattering point Q to the radar can be approximated as:

(6) $$r(t)≅R_{m}(t)+R_{p}(t)+x_{n}-y_{n}\omega t$$

Therefore, the backscattered echoes from all the scatterers can be theoretically be represented as:

(7) $$\begin{array}{ll} E^{s}(r,t) & =\sum_{n=1}^{N}A_{n}\cdot e^{-j2kr(t)}\\ & =\sum_{n=1}^{N}A_{n}\cdot e^{-j2k\left(R_{m}(t)+R_{p}(t)+x_{n}-y_{n}\omega t\right)}\\ & =\sum_{n=1}^{N}A_{n}\cdot e^{-j2k(R_{0}+x_{n})}\cdot e^{-j2k\left(v_{t}+\frac{1}{2}a_{t}t^{2}\right)}\cdot e^{-j2k\left(v_{p}+\frac{1}{2}a_{p}t^{2}\right)}\cdot e^{j2k(y_{n}\omega t)} \end{array}$$

Here k=2πf/c. c represents the electromagnetic wave speed and $A_{n}$ is the scattering intensity.

The Doppler shift induced by the target motion and radar platform displacement can be obtained by time derivative of Equation (6):

(8) $$f_{d}{}_{1}=-\frac{1}{2\pi }\left(-\frac{4\pi f}{c}\cdot \frac{dr(t)}{dt}\right)=\frac{2f}{c}[(v_{t}+v_{p}-y_{n}\omega )+(a_{t}+a_{p})t]$$

From (7), we can get the maneuvering target’s magnitude:

(9) $$\begin{array}{ll} \left|E^{s}(k,t)\right| & ≅\left|\sum_{n=1}^{N}A_{n}\cdot e^{-j2k(R_{0}+x_{n})}\cdot e^{-j2k\left(v_{t}+\frac{1}{2}a_{t}t^{2}\right)}\cdot e^{-j2k\left(v_{p}+\frac{1}{2}a_{p}t^{2}\right)}\cdot e^{j2k(y_{n}\omega t)}\right|\\ & =\left|\sum_{n=1}^{N}A_{n}\cdot e^{-j2k(R_{0}+x_{n})}\cdot e^{j2k(y_{n}\omega t)}\right|\left|e^{-j2k\left(v_{t}+\frac{1}{2}a_{t}t^{2}\right)}\right|\left|e^{-j2k\left(v_{p}+\frac{1}{2}a_{p}t^{2}\right)}\right|\\ & =\left|\sum_{n=1}^{N}A_{n}\cdot e^{-j2k(R_{0}+x_{n})}\cdot e^{j2k(y_{n}\omega t)}\right| \end{array}$$

As can be seen from the Equation above, the ISAR echo module is not affected by the radial displacement of the aerostat-borne radar. Therefore, ISAR autofocus imaging can be achieved by implementing phase retrieval algorithm under the condition that the radar platform is unstable.

### 2.2. ISAR Echo Analysis of Platform Fluctuation

In addition to the platform displacement, the radar platform will experience a small amplitude of vibration due to the airflow. The traditional ISAR imaging technology is based on the motion parameters of the target, but the randomness of the platform vibration will bring more difficulties to the traditional ISAR imaging method. With respect to the radar geometry model shown in Figure 1, the echo of the radial vibration of the radar platform is analyzed. When the radar platform vibration occurs, the expression of *R_p_*_1_(*t*) in Equation (4) will change:

(10) $$R_{p_{1}}(t)=L\sin2\pi f_{vib}t$$

where L and $f_{vib}$ are respectively the amplitude and frequency of the radar platform vibration.

The distance $r_{1}(t)$ at this stage from the scattering point *Q* to the radar can be approximated as:

(11) $$r_{1}(t)≅R_{m}(t)+R_{p_{1}}(t)+x_{n}-y_{n}\omega t$$

Therefore, the backscattered echoes from all the scatterers can be written as follows:

(12) $$\begin{array}{ll} {E^{s}}_{1}(r,t) & =\sum_{n=1}^{N}A_{n}\cdot e^{-j2kr_{1}(t)}\\ & =\sum_{n=1}^{N}A_{n}\cdot e^{-j2k\left(R_{m}(t)+R_{p_{1}}(t)+x_{n}-y_{n}\omega t\right)}\\ & =\sum_{n=1}^{N}A_{n}\cdot e^{-j2k(R_{0}+x_{n})}\cdot e^{-j2k\left(v_{t}+\frac{1}{2}a_{t}t^{2}\right)}\cdot e^{-j2k(L\sin2\pi f_{vib}t)}\cdot e^{j2k(y_{n}\omega t)} \end{array}$$

According to the above equation, we can obtain its frequency shift:

(13) $$f_{d2}=-\frac{1}{2\pi }\left[-\frac{4\pi f}{c}\cdot \frac{dr_{1}(t)}{dt}\right]=\frac{2f}{c}[(v_{t}-y_{n}\omega )+2\pi Lf_{vib}\cdot \cos2\pi f_{vib}t+a_{t}t]$$

The maneuvering target’s magnitude can be represented as:

(14) $$\begin{array}{ll} \left|E^{s}{}_{1}(k,t)\right| & ≅\left|\sum_{n=1}^{N}A_{n}\cdot e^{-j2k(R_{0}+x_{n})}\cdot e^{-j2k\left(v_{t}+\frac{1}{2}a_{t}t^{2}\right)}\cdot e^{-j2k(L\sin2\pi f_{vib}t)}\cdot e^{j2k(y_{n}\omega t)}\right|\\ & =\left|\sum_{n=1}^{N}A_{n}\cdot e^{-j2k(R_{0}+x_{n})}\cdot e^{j2k(y_{n}\omega t)}\right|\left|e^{-j2k\left(v_{t}+\frac{1}{2}a_{t}t^{2}\right)}\right|\left|e^{-j2k(L\sin2\pi f_{vib}t)}\right|\\ & =\left|\sum_{n=1}^{N}A_{n}\cdot e^{-j2k(R_{0}+x_{n})}\cdot e^{j2k(y_{n}\omega t)}\right| \end{array}$$

Seen from the above equation, the ISAR echo module is still not affected by the vibration of the aerostat-borne radar. Therefore, the method proposed in this paper can handle the difficulties of motion compensation in the above two cases.

## 3. Non-Stationary Platform ISAR Imaging Based on Improved Phase Retrieval Algorithm

### 3.1. Phase Retrieval Principle

In the field of optical imaging, an imaged object is illuminated by a laser beam and projected onto an optical detection device, and its far field and near field Fourier transform are the same for an electromagnetic field. Therefore, once the Fourier magnitude and phase value of the far field are known, the original target imaging result can be obtained. However, since the electromagnetic oscillation frequency of the light quantum is often greater than 10^15^ Hz, the phase information cannot be directly recorded in the process of converting to electrons. The optical device first needs to measure the photon flux that is proportional to the Fourier amplitude spectrum of the imaged object, and then the phase retrieval algorithm is carried out for imaging. As a popular imaging technology, Coherent Diffraction Imaging (CDI) is a method that combines X-ray diffraction, oversampling and phase retrieval.

It is supposed that one-dimensional discrete real field distribution function of an object is $x\in C^{N}$, and its one-dimensional discrete Fourier transform can be expressed as:

(15) $$X(k)=\sum_{n=0}^{N-1}x(n)e^{-j2\pi \frac{kn}{M}}\;k=0,1,\ldots ,M-1$$

where M represents the M-point discrete Fourier transform of the function, and M>N. We rewrite the equation as:

(16) $$X\left(k\right)=\left|X\left(k\right)\right|e^{j\phi (k)}\;k=0,1,\ldots ,M-1$$

The principle of the phase retrieval algorithm is to recover the Fourier phase information ϕ(k) using only the known Fourier transform information |X(k)|, then the distribution function $\hat{x}$ is recovered by performing the inverse Fourier transform.

### 3.2. OSS Phase Retrieval Algorithm

**Figure 2:**
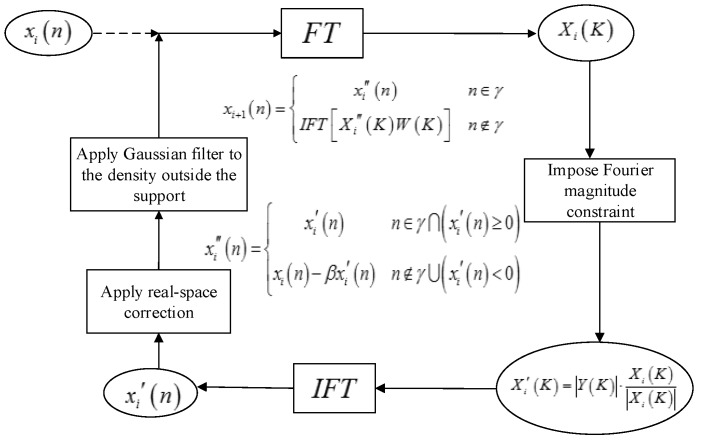
The schematic of the OSS phase retrieval algorithm.

To solve the problem of retrieval when the initial input Fourier amplitude spectrum is disturbed and mixed with noise, the OSS algorithm [27] adds iterative steps of frequency domain filtering after the support domain constraints of the traditional HIO algorithm [29]. Figure 2 shows the process of the algorithm from the ith to the (i+1)th iteration at each run.

1. $x_{i}(n)$ is the signal to be recovered with initial random phase. Obtain a Fourier pattern $X_{i}(K)$ by performing the Fourier transform to $x_{i}(n)$.
2. Retain the phase information of $X_{i}(K)$, but replace the magnitude of $X_{i}(K)$ with the known Fourier intensity |Y(K)| to generate a new complex-valued function $X_{i}^{\prime }(K)$, where |Y(K)| is the magnitude of the measured ISAR echo signal.
3. Perform an inverse Fourier transform on $X_{i}^{\prime }(K)$ to generate a new image $x_{i}^{\prime }(n)$. Revise $x_{i}^{\prime }(n)$ on the basis of HIO algorithm and get a new $x_{i}^{\prime \prime }(n)$.
  (17) $$x_{i}^{\prime \prime }\left(n\right)=\left\{ \begin{array}{cc} x_{i}^{\prime }\left(n\right) & n\in \gamma \cap \left(x_{i}^{\prime }\left(n\right)\geq 0\right)\\ x_{i}\left(n\right)-\beta x_{i}^{\prime }\left(n\right) & n\notin \gamma \cup \left(x_{i}^{\prime }\left(n\right)<0\right) \end{array}\right.$$
  where γ represents a finite support and β is a parameter between 0.5 and 1.
4. Calculate the next iteration image $x_{i+1}(n)$:
  (18) $$x_{i+1}\left(n\right)=\{\begin{array}{cccc} x_{i}^{\prime \prime }\left(n\right) & n\in \gamma & & \\ IFT\left[X_{i}^{\prime \prime }\left(K\right)W\left(K\right)\right] & n\notin \gamma & & \end{array}$$
  where $X_{i}^{\prime \prime }(K)$ represents the Fourier pattern of $x_{i}^{\prime \prime }(n)$. W(K) is a normalized Gaussian function in Fourier domain, which is defined as:
  (19) $$W(K)=\exp[-\frac{1}{2}{\left(K/\partial \right)}^{2}]$$

The smoothing filter W(K) is only applied the density outside the support domain. The width of the Gaussian filter can be adjusted to handle the impact of high-frequency information outside the support by changing parameter ∂.

### 3.3. ISAR Autofocus Imaging Method Based on Improved Phase Retrieval

From the analysis of Section 2.1 and Section 2.2, the relative motion between the target and the aerostat borne radar platform will add error phases to the original ideal signal. Assuming $\tilde{E^{s}}(k,t)$ is an ideal echo signal with the phase error $\psi_{e}(k,t)$ induced by the platform’s motion and target’s motion, the actual signal received can be expressed as:

(20) $$E^{s}(k,t)=\tilde{E^{s}}(k,t)\cdot e^{j\psi_{e}(k,t)}$$

From the above equation, we can see that the radial motion of the radar platform does not have a negative effect on the amplitude of the received signal. In this way, the amplitude in Equation (20) is the same as that of the echo when the radar platform is stationary, which is the theoretical basis for phase retrieval algorithm to perform motion compensation.

**Figure 3:**
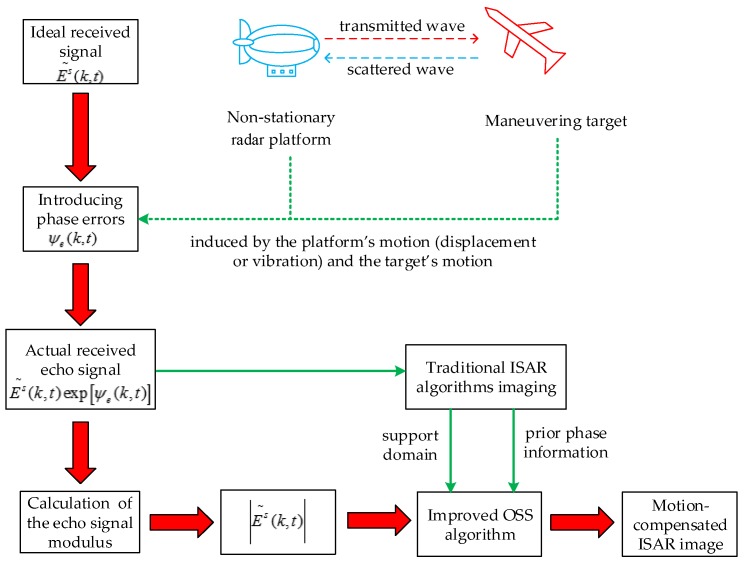
Block scheme of the proposed method.

It should be pointed out that the classical phase retrieval algorithm tends to have a lower success rate if only the echo module information is used to recover the ISAR image. Moreover, the correctness of the recovery result cannot be guaranteed due to the lack of a priori information. Therefore, we propose an improved OSS phase retrieval algorithm that utilizes a priori information (or error information), that is, the phase of the blurred image obtained by the classical imaging algorithm (such as RD algorithm, cross-correlation method, etc.) replaces the initial random phase in the original OSS algorithm. Besides, the support domain size of OSS algorithm is set with respect to the blurred target image. The block scheme of this algorithm is shown in Figure 3.

## 4. Simulation Analysis

To verify the validity of the approach proposed in this paper, we conducted three sets of experiments. Table 1 shows the radar parameters and the target motion parameters used in the simulation. In Section 4.1 and Section 4.2, to depict the resultant range Doppler ISAR image under the conditions that the motion of aerostat-borne radar platform occurs, the radar parameters and the target motion parameters in Table 1 [30] remain unchanged, and the imaging results obtained by the popular algorithms and the proposed method are compared only by changing the radar platform motion parameters.

### 4.1. Imaging Results with Different Radial Displacements of Radar Platform

**Figure 4:**
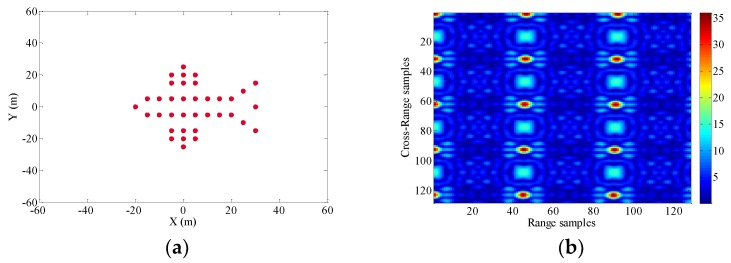
(**a**) A hypothetical target composed of perfect point scatterers. (**b**) The amplitude of the raw data.

The hypothetical airplane composed of point scatterers and the ISAR echo signal modulus under the condition of a stable aerostat borne radar platform are shown in Figure 4. It is assumed that the radar platform has a radial velocity $v_{p}$ and a radial acceleration $a_{p}$.

**Figure 5:**
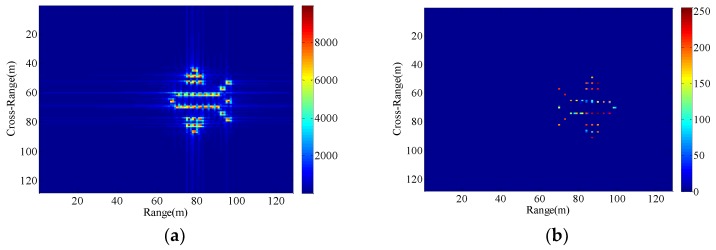
Imaging results with the radar platform displacement parameters P1. (**a**) RD algorithm; (**b**) the proposed method.

**Figure 6:**
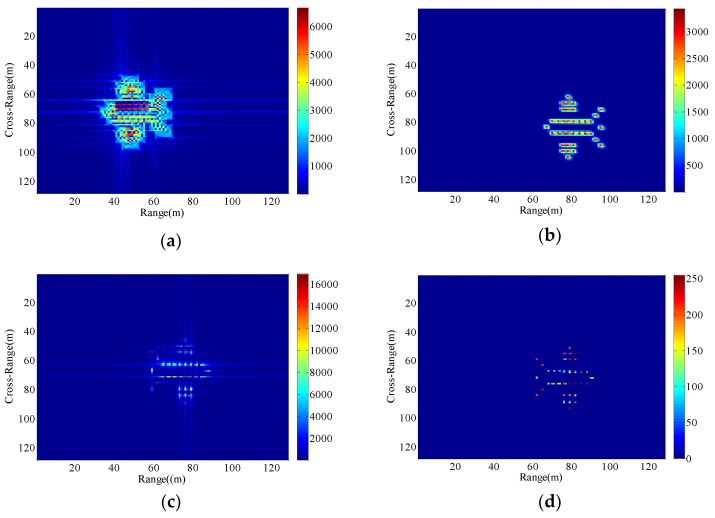
Imaging results with the radar platform displacement parameters P2. (**a**) RD algorithm; (**b**) cross-correlation method; (**c**) minimum entropy method; (**d**) the proposed method.

**Figure 7:**
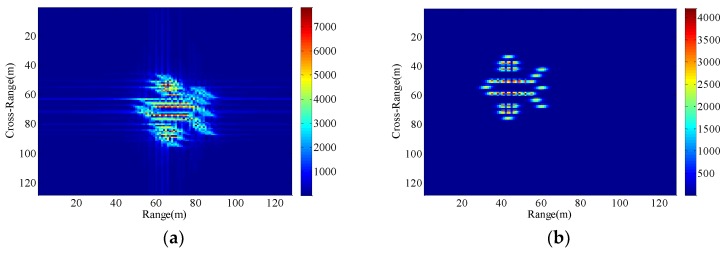

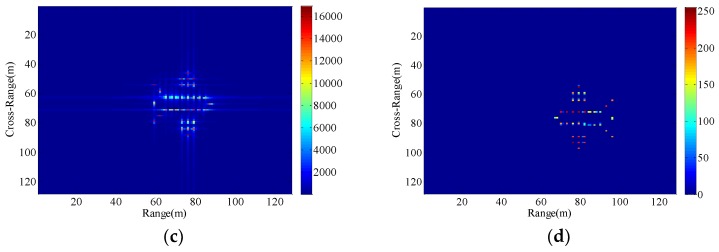
Imaging results with the radar platform displacement parameters P3. (**a**) RD algorithm; (**b**) cross-correlation method; (**c**) minimum entropy method; (**d**) the proposed method.

**Figure 8:**
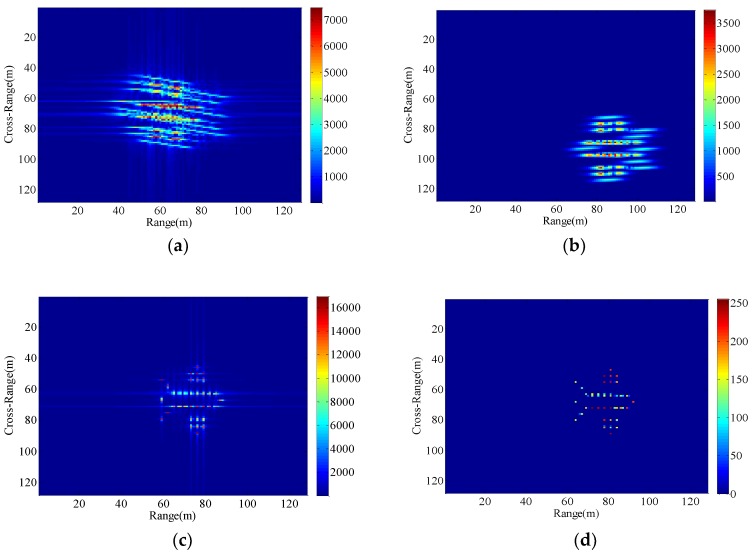
Imaging results with the radar platform displacement parameters P4. (**a**) RD algorithm; (**b**) cross-correlation method; (**c**) minimum entropy method; (**d**) the proposed method.

Consequently, The ISAR images with different radial displacements of radar platform are obtained as shown in Figure 5, Figure 6, Figure 7 and Figure 8 by applying the RD algorithm, cross-correlation method, minimum entropy method and the phase retrieval algorithm proposed in this paper.

Different parameters from the experiments are listed in Table 2 for a more intuitive view.

As can be seen from Figure 5a, RD algorithm can be applied to target imaging with slow motion under the condition of a stationary aerostat-borne radar platform. However, RD algorithm is no longer applicable when radial motion of the radar platform occurs due to the air flow effects. The cross-correlation method can only estimate a fixed radial velocity within a preset interval, so when the radial velocity becomes large and the radial acceleration is small, Figure 6b clearly demonstrates the success of the radial velocity compensation such that only the acceleration-based defocusing is noted in the ISAR image. When the radial velocity becomes smaller and the radial acceleration is slightly larger, the resulting image is depicted in Figure 7b and Figure 8b where the image is highly distorted because of the large errors in the parameter estimation of the cross-correlation method. Compared with cross-correlation method, although minimum entropy method has a better performance in the parameter estimation the minimum entropy method can not only estimate the velocity value but also the acceleration value in a certain range, it must be set an appropriate search range and step length first, otherwise it is not possible to perfectly image the target scatterings. From Figure 6c, Figure 7c, and Figure 8c, the dominant motion effects of translational motion are successfully eliminated by the minimum entropy method, but the proposed method in this paper outperforms the minimum entropy method in Figure 6d, Figure 7d, and Figure 8d.

The echo modules with the different radial motion parameters of radar platform in Figure 6, Figure 7 and Figure 8 are unchanged, which is the same as the ISAR echo module in Figure 5. It is verified that the radial motion of the radar platform does not affect the echo module, and the proposed method can be used for ISAR autofocus imaging.

**Figure 9:**
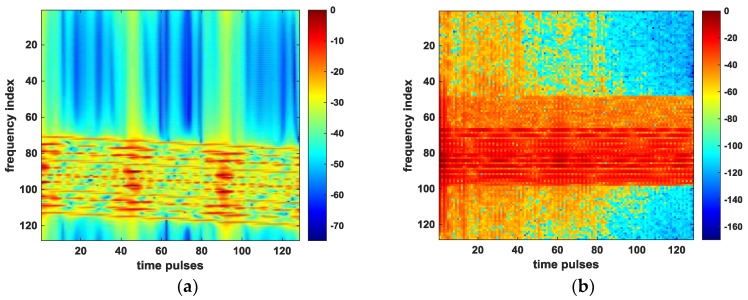
Spectrograms of time pulses. (**a**) before applying the proposed method; (**b**) after applying the proposed method.

The further check is performed by looking at the spectrogram of the received time pulses with respect to Figure 8a,d, which can reflect the change in frequency shift in Equation (8). We can see from Figure 8a that before the OSS phase retrieval algorithm is applied, the severe frequency shifts due to the target motion and radar platform motion have occurred in Figure 9a. After compensating for the errors associated with target’s motion by using OSS phase retrieval, these shifts are well aligned, as shown in Figure 9b.

### 4.2. Imaging Results with Different Radar Platform Vibration Parameters

**Figure 10:**
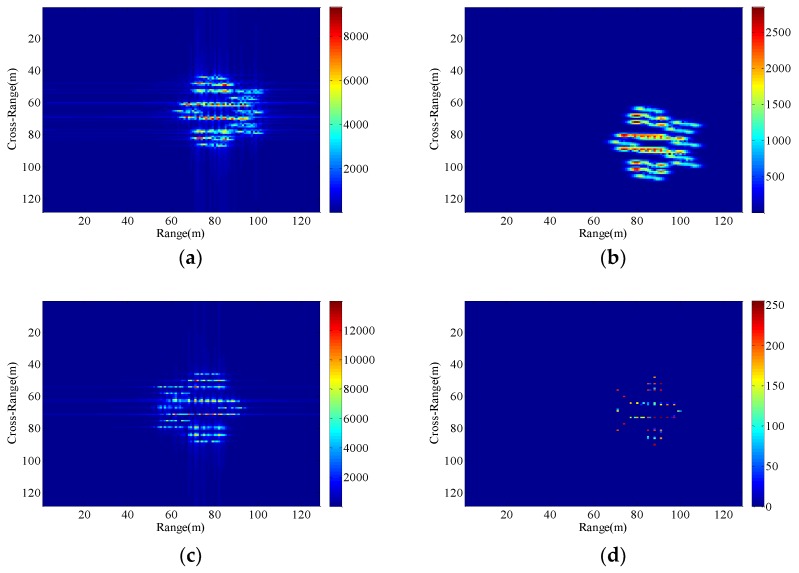
Imaging results with the radar platform vibration parameters P5. (**a**) RD algorithm; (**b**) cross-correlation method; (**c**) minimum entropy method; (**d**) the proposed method.

**Figure 11:**
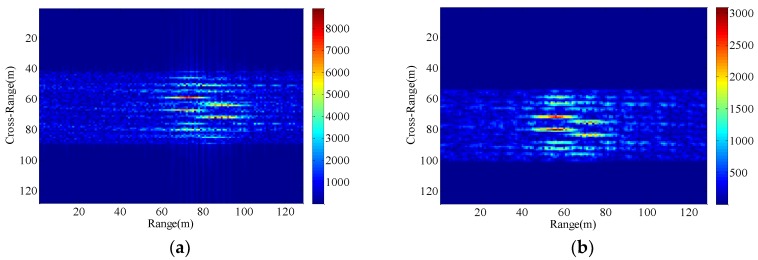

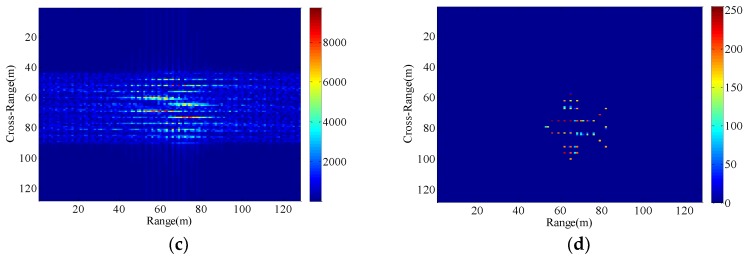
Imaging results with the radar platform vibration parameters P6. (**a**) RD algorithm; (**b**) cross-correlation method; (**c**) minimum entropy method; (**d**) the proposed method.

**Figure 12:**
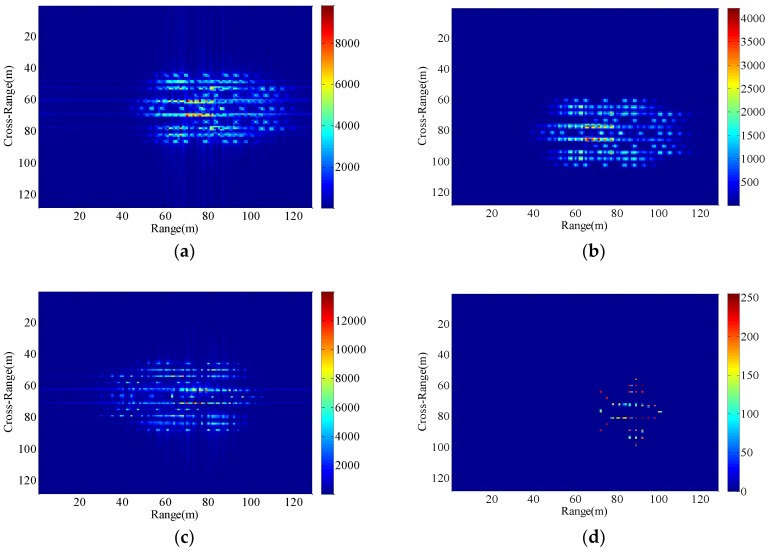
Imaging results with the radar platform vibration parameters P7. (**a**) RD algorithm; (**b**) cross-correlation method; (**c**) minimum entropy method; (**d**) the proposed method.

We set up three sets of comparative experiments with different vibration parameters, and the resultant images are shown in Figure 10, Figure 11 and Figure 12. As is obvious from Figure 10, the uncompensated ISAR image is highly distorted and blurred. Compared with Figure 10, the vibration frequency of the radar platform in Figure 11 remains unchanged and the vibration amplitude is larger. Due to the increase in vibration amplitude, the effect of target’s vibration is severe in the Figure 11a–c. Different from Figure 10, the vibration amplitude of the radar platform in Figure 12 remains unchanged and the vibration frequency is increased. In this case, the resultant ISAR images obtained by the traditional algorithm are broadly blurred in the range and Doppler domains where the higher the vibration frequency is, the more serious the overlap will be. From the analysis of Section 4.1, although the minimum entropy method and the cross-correlation method can remove the effects of radial displacement motion in the case of small radial translation velocity and acceleration, none of them can overcome the issues of the radar platform vibration. Since the radial vibration of the radar platform does not affect the echo module, the proposed method can eliminate the unwanted effects due to target’s vibration.

**Figure 13:**
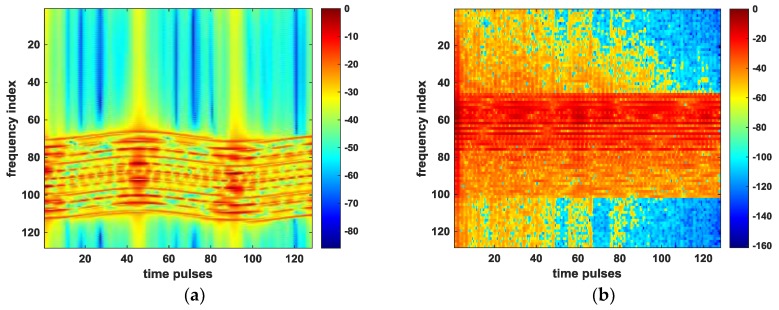
Spectrograms of time pulses. (**a**) before applying the proposed method; (**b**) after applying the proposed method.

The spectrograms of the time pulses in the received signal are also plotted in Figure 13 with respect to Figure 11a,d. By analyzing the Doppler shift in this case (obtained from Equation (13)), we find that since the rotation component of the target is small, the frequency shifts are mainly caused by the platform vibration and the target motion, so there exists significant fluctuation due to platform vibration in the frequency of time pulses in Figure 13a. As is obvious from Figure 13b, all frequency values of the returned pulses are aligned successfully, which proves the good performance of the proposed method under the condition of the radar platform vibration.

### 4.3. Imaging Results of the Proposed Method with Different Target Motion Parameters under the Condition of Non-Stationary Radar Platform

**Figure 14:**
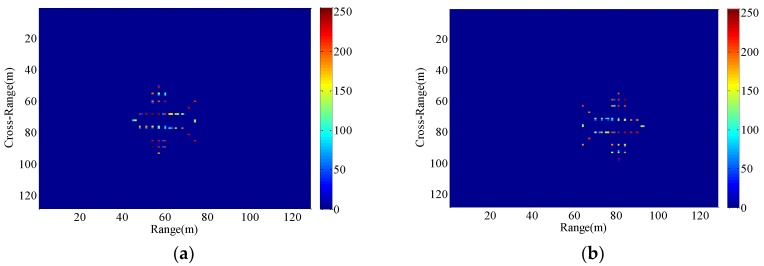
Imaging results with the radar platform displacement parameters P8. (**a**) P9; (**b**) P10.

**Figure 15:**
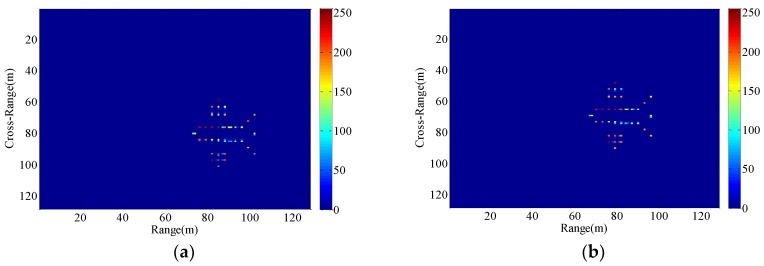
Imaging results with the radar platform vibration parameters P11. (**a**) P12; (**b**) P13.

The motion parameters of the maneuvering target in Section 4.1 and Section 4.2 are fixed, and only the motion parameters of the radar platform are changing. In order to demonstrate the ISAR imaging results of the proposed method with different radial motion parameters under the condition of unstable radar platform, another set of experiment were carried out. As can be seen from Figure 14 and Figure 15, the resultant ISAR images are clear and focused in both range and cross-range directions, verifying that the proposed method can perform autofocus imaging of the target with different motion parameters.

## 5. Conclusions

In this paper, a phase retrieval method for aerostat-borne ISAR autofocus imaging has been proposed. In general, the radial displacement and radial vibration of the radar platform due to airflow will affect the stability of the radar platform, making the range-Doppler ISAR image highly defocused and blurred. Based on the aerostat-borne ISAR imaging geometry model, we can deduce that ISAR echo module is not affected by the radial displacement and the vibration of the aerostat borne radar under the condition of the moving maneuvering target. Therefore, combined with classic OSS phase retrieval algorithm and the prior phase information that the traditional ISAR imaging technology can provide, we theoretically prove that the proposed method can overcome the difficulties of motion compensation in the above cases.

In the experimental simulation, we compare the imaging results of the RD algorithm, cross-correlation method, minimum entropy method with the imaging results of the proposed method. The former three traditional methods cannot successfully eliminate the motion effects of radar platforms and maneuvering targets. The method can obtain resultant motion-free ISAR image after completely removing the phase error of the received signal, wherein the scattering centers around the target are well localized. Additionally, we also show some imaging results of the proposed method with different target motion parameters under the condition of quasi-stationary radar platform, which further expand the application conditions of this method.

In summary, the results of this study provide a new way of thinking for the non-stationary platform ISAR imaging problem. Of course, it is very important that the algorithm does not estimate any relevant motion parameters. The future work will focus on a new approach for fast autofocus imaging, where the convolutional neural network is applied to recover the original phase of the radar received signal.

## Figures and Tables

**Table 1:** The radar parameters for SFCW (Stepped frequency continuous wave) illumination.

Parameter Name	Symbol	Value
Target’s initial position in range	*R*_0_	16 km
Starting frequency	*f*_0_	9 GHz
Frequency bandwidth	*B*	125 MHz
Pulse repetition frequency	*PRF*	35 KHz
Number of pulses	*N_pulse_*	128
Number of bursts	*M_burst_*	128
Target radial velocity	*v_t_*	5 m/s
Target radial acceleration	*a_t_*	0.06 m/s^2^
Target’s rotational velocity	ω	0.02 rad/s

**Table 2:** The motion parameters of the radar platform and maneuvering target in different experiments.

Symbol	Parameter Value
P1	*v_p_* = 0 m/s, *a_p_* = 0 m/s^2^
P2	*v_p_* = 17 m/s, *a_p_* = 0.06 m/s^2^
P3	*v_p_* = 13 m/s, *a_p_* = 0.6 m/s^2^
P4	*v_p_* = 8 m/s, *a_p_* = 1.94 m/s^2^
P5	*L* = 0.01m, *f_vb_* = 3 Hz
P6	*L* = 0.5m, *f_vb_* = 3 Hz
P7	*L* = 0.01m, *f_vb_* = 10 Hz
P8	*v_p_* = 25 m/s, *a_p_* = 0.2 m/s^2^
P9	*v_t_* = 10 m/s, *a_t_* = 0.5 m/s^2^
P10	*v_t_* = 20 m/s, *a_t_* = 1 m/s^2^
P11	*L* = 1m, *f_vb_* = 20 Hz
P12	*v_t_* = 10 m/s, *a_t_* = 0.5 m/s^2^
P13	*v_t_* = 20 m/s, *a_t_* = 1 m/s^2^

## References

[1] Hu J., Zhang J., Zhai Q., Zhan R., Lu D. (2014). ISAR imaging using a new stepped-frequency signal format. IEEE Trans. Geosci. Remote Sens..

[2] Zhou X., Wei G., Wu S., Wang D. (2016). Three-dimensional ISAR imaging method for high-speed targets in short-range using impulse radar based on SIMO array. Sensors.

[3] Shi H., Xia S. ISAR imaging based on oversampling smoothness of prior knowledge. Proceedings of the 2016 IEEE 13th International Conference on Signal Processing (ICSP).

[4] Zheng J., Liu H., Liao G., Su T., Liu Z., Liu Q.H. (2016). ISAR imaging of targets with complex motions based on a noise-resistant parameter estimation algorithm without nonuniform axis. IEEE Sens. J..

[5] Yong W., Abdelkader A.C., Zhao B., Wang J. (2015). ISAR Imaging of Maneuvering Targets Based on the Modified Discrete Polynomial-Phase Transform. Sensors.

[6] Wang Y. (2015). Radar Imaging of Non-Uniformly Rotating Targets via a Novel Approach for Multi-Component AM-FM Signal Parameter Estimation. Sensors.

[7] Vierling L.A., Fersdahl M., Chen X., Li Z., Zimmerman P. (2006). The Short Wave Aerostat-Mounted Imager (SWAMI): A novel platform for acquiring remotely sensed data from a tethered balloon. Remote Sens. Environ..

[8] Liu S., Niu Z., Wu Y. (2004). A Blockage based Channel Model for High Altitude Platform Communications. Chin. J. Electron..

[9] Fan L., Shi S., Liu Y., Xu S., Chen Z. (2015). A novel Range-Instantaneous-Doppler ISAR imaging algorithm for maneuvering targets via adaptive doppler spectrum extraction. Prog. Electromagnet. Res. C..

[10] Wang Y., Lin Y. (2013). ISAR Imaging of Non-Uniformly Rotating Target via Range-Instantaneous-Doppler-Derivatives Algorithm. IEEE J. Sel. Topics Appl. Earth Observ. Remote Sens..

[11] Mateus P., Nico G., Tome R., Catalao J. (2013). Experimental Study on the Atmospheric Delay Based on GPS, SAR Interferometry, and Numerical Weather Model Data. IEEE Trans. Geosci. Remote Sens..

[12] Karakasiliotis A.V., Lazarov A.D., Frangos P.V., Boultadakis G., Kalognomos G. (2008). Two-dimensional ISAR model and image reconstruction with stepped frequency-modulated signal. IET Signal Process..

[13] Suwa K., Wakayama T., Iwamoto M. (2011). Three-dimensional target geometry and target motion estimation method using multistatic ISAR movies and its performance. IEEE Trans. Geosci. Remote Sens..

[14] Ustun D., Ozdemir C., Akdagli A., Toktas A., Bicer M.B. (2014). A powerful method based on artificial bee colony algorithm for translational motion compensation of ISAR image. Microw. Opt. Technol. Lett..

[15] Xue J., Huang L. An improved cross-correlation approach to parameter estimation based on fractional Fourier transform for ISAR motion compensation. Proceedings of the 2015 IEEE International Conference on Acoustics, Speech and Signal Processing (ICASSP).

[16] Zhang S., Liu Y., Li X. (2015). Fast entropy minimization based autofocusing technique for ISAR imaging. IEEE Trans. Signal Process..

[17] Shin S.Y., Myung N.H. (2008). The application of motion compensation of ISAR image for a moving target in radar target recognition. Microwave Opt. Tech. Lett..

[18] Li D., Zhan M., Liu H., Liao G., Liao Y. (2017). A Robust Translational Motion Compensation Method for ISAR Imaging Based on Keystone Transform and Fractional Fourier Transform under Low SNR Environment. IEEE Trans. Aerosp. Electron. Syst..

[19] Chen V.C., Martorella M. (2014). Inverse Synthetic Aperture Radar Imaging: Principles, Algorithms and Applications.

[20] QI Z., Jing Y., You S.Q., Sun H.B. (2013). The quasi-stationary platform of stratospheric ISAR imaging. J. China Univ. Posts Telecommun..

[21] Shechtman Y., Eldar Y.C., Cohen O., Chapman H.N., Miao J., Segev M. (2014). Phase Retrieval with Application to Optical Imaging: A contemporary overview. IEEE Signal Process. Mag..

[22] Marchesini S., He H., Chapman H.N., Hauriege S.P., Noy A., Howells M.R., Weierstall U., Spence J.C.H. (2003). X-ray image reconstruction from a diffraction pattern alone. Phys. Rev. B.

[23] Fannjiang A. (2012). Absolute uniqueness of phase retrieval with random illumination. Inverse Probl..

[24] Shenoy B.A., Mulleti S., Seelamantula C.S. (2016). Exact phase retrieval in principal shift-invariant spaces. IEEE Trans. Signal Process..

[25] Netrapalli P., Jain P., Sanghavi S. (2015). Phase retrieval using alternating minimization. IEEE Trans. Signal Process..

[26] Takajo H., Takahashi T., Ueda R., Taninaka M. (1998). Further study on the convergence property of the hybrid input–output algorithm used for phase retrieval. J. Opt. Soc. Am. A.

[27] Rodriguez J.A., Xu R., Chen C.C., Zou Y., Miao J. (2013). Oversampling smoothness: An effective algorithm for phase retrieval of noisy diffraction intensities. J. Appl. Crystallogr..

[28] Schniter P., Rangan S. (2015). Compressive phase retrieval via generalized approximate message passing. IEEE Trans. Signal Process..

[29] Chen C.C., Miao J., Wang C.W., Lee T.K. (2007). Application of optimization technique to noncrystalline X-ray diffraction microscopy: Guided hybrid input-output method. Phys. Rev. B.

[30] Ozdemir C. (2012). Inverse Synthetic Aperture Radar Imaging with MATLAB Algorithms.

